# Muscle synergies are associated with intermuscular coherence and cortico-synergy coherence in an isometric upper limb task

**DOI:** 10.1007/s00221-023-06706-6

**Published:** 2023-09-22

**Authors:** Pablo Ortega-Auriol, Winston D. Byblow, Thor Besier, Angus J. C. McMorland

**Affiliations:** 1 Department of Exercise Sciences, University of Auckland, Auckland, New Zealand; 2 Centre for Brain Research, University of Auckland, Auckland, New Zealand; 3 Auckland Bioengineering Institute, The University of Auckland, Auckland, New Zealand

**Keywords:** Muscle synergies, Coherence, Functional connectivity, Electromyography, Coordination, Upper limb

## Abstract

To elucidate the underlying physiological mechanisms of muscle synergies, we investigated long-range functional connectivity by cortico-muscular (CMC), intermuscular (IMC) and cortico-synergy (CSC) coherence. Fourteen healthy participants executed an isometric upper limb task in synergy-tuned directions. Cortical activity was recorded using 32-channel electroencephalography (EEG) and muscle activity using 16-channel electromyography (EMG). Using non-negative matrix factorisation (NMF), we calculated muscle synergies from two different tasks. A preliminary multidirectional task was used to identify synergy-preferred directions (PDs). A subsequent coherence task, consisting of generating forces isometrically in the synergy PDs, was used to assess the functional connectivity properties of synergies. Overall, we were able to identify four different synergies from the multidirectional task. A significant alpha band IMC was consistently present in all extracted synergies. Moreover, IMC alpha band was higher between muscles with higher weights within a synergy. Interestingly, CSC alpha band was also significantly higher across muscles with higher weights within a synergy. In contrast, no significant CMC was found between the motor cortex area and synergy muscles. The presence of a shared input onto synergistic muscles within a synergy supports the idea of neurally derived muscle synergies that build human movement. Our findings suggest cortical modulation of some of the synergies and the consequential existence of shared input between muscles within cortically modulated synergies.

## Introduction

The musculoskeletal system is highly redundant, meaning many possible muscle activations can achieve the same single purposeful action. Dimensionality reduction applied to EMG signals from multiple muscles shows the presence of lower dimensional networks, which we call muscle synergies. These low-dimensional networks can explain the behaviour of the complete set of muscles measured. There are two ongoing debates relating to muscle synergies that we will address in this study. First, do muscle synergies represent a deliberate neurophysiological control strategy (Bizzi and Cheung 2013; McMorland et al. 2015) or are they merely an artefact of the task requirements and mathematical derivation (Kutch and Valero-Cuevas 2012). Second, if muscle synergies arise from a control strategy, which neural structures are responsible for their emergence and modulation?

Evidence exists on both sides of the debate on whether synergies arise from neural structures. Animal studies using electrical stimulation (Tresch and Bizzi 1999; Bizzi et al. 2008; Hart and Giszter 2013) and computational (Neptune et al. 2009) models support a neural origin for muscle synergies. Similarly, human experiments are consistent with a neural source of muscle synergies during natural movements (d’Avella and Bizzi 2005; Safavynia and Ting 2012), affecting learning rates (Berger et al. 2013; Sawers et al. 2015), for postural control (Weiss and Flanders 2004), when extracted from the frequency domain (Frere 2017), irrespective of muscle fatigue (Ortega-Auriol et al. 2018), and in the presence of CNS damage after stroke (Cheung et al. 2009; Berger et al. 2013). Conversely, evidence for muscle synergies due to purely mechanical constraints arises from computer simulations of the upper arm movement on a single plane (Inouye and Valero-Cuevas 2016) and cadaveric studies (Kutch and Valero-Cuevas 2012). While it seems possible that muscle synergies may arise from both neural and mechanical mechanisms, there are still good reasons to determine which neural structures are implicated in their expression.

A viable theory about movement control must contain a neuroanatomical framework capable of discerning the origin of muscle synergies (McMorland et al. 2015). Movement control can be deconstructed into not only three primary sources of drive, but also the contributions of complex neural loops, including deeper brain structures like the basal ganglia and thalamus, as well as brainstem and cerebellum. The original three sources of drive: cortical activity, spinal activity through central pattern generators, and adjustment reflexes remain integral to the process, but acknowledging the potential involvement of other neural structures enriches our understanding of muscle synergy encoding (Ivanenko et al. 2005). A few experimental approaches aim to differentiate between the possible anatomical neural origin of motor behaviours. Coherence, a non-directional signal frequency-based analysis, is proposed to identify common functional control sources across muscles during a motor task (Laine and Valero-Cuevas 2017). Coherence is a correlation measure between two signals in a determined frequency band (Boonstra 2009). Different factors such as cortical spectral power (Kristeva et al. 2007) and sensory feedback (Fisher et al. 2002) can modulate coherence levels. Cortico-muscular coherence (CMC) between brain (EEG) and muscle (EMG) activity occurs around the beta band (15–30 Hz), suggestive of cortical control of movement (Conway et al. 1995; Baker et al. 2003; Gwin and Ferris 2012). Intermuscular coherence (IMC), between EMG of two different muscles, occurs around the ~ 10 Hz or alpha band (Boonstra 2009) and is considered to reflect subcortical control (Boonstra 2009; Marchis et al. 2015). Recently, a few studies have proposed cortico-synergy coherence (CSC) as a measure of connectivity between a muscle synergy’s activation coefficient and EEG from the motor cortex (Zandvoort et al. 2019, 2021). Coherent activity between two signals suggests common neurophysiological sources of neural drive or a long-range synchronisation network (Aumann and Prut 2015).

On a functional level, CMC, CSC and IMC are measures that can provide insight into the underlying coordination of a motor task, differentiating pathways converging onto spinal motor neurons, and identifying shared information across different scattered muscles (Boonstra 2013). The synchronisation of oscillatory activity across the neuromuscular system, measured by coherence, reflects the necessary functional coupling to control movement. For example, IMC in the α band across muscles or even bilateral muscles suggests a common input from subcortical structures (Farmer et al. 1993; Boonstra 2009) and may reflect the involvement of the reticulospinal pathway (Grosse and Brown 2003). Synchronisation within the beta band during precise, steady force outputs of hand muscles has been proposed to indicate functional binding between the primary motor cortex and effector muscles (Kilner et al. 2000; Boonstra 2009; Danna-Dos Santos et al. 2010). While CMC and IMC are well-established measurements, CSC is relatively new with limited supporting evidence. However, based on previous findings related to CMC and IMC, it is possible to speculate about the implications of CSC. Further research is needed to fully comprehend and validate these implications.

IMC activity has been described across muscles of an individual synergy during postural responses (Danna-Dos-Santos et al. 2014) and a cycling task (Marchis et al. 2015). However, there was little evidence for cortical involvement, given that beta or gamma IMC was observed in only a single synergy of the many extracted. These findings suggest a synergy’s origin from a biomechanically constrained task or a non-neural origin, considering the non-significant levels of coherence during the tasks. At the same time, a single functional task might not be an optimal paradigm to determine coherence within synergies, given the possibility of a biomechanically constrained synergy and lack of space volume exploration. Instead, motor tasks tuned to the preferred direction of a synergy (Ortega-Auriol et al. 2018) that preferentially recruit a single synergy could be a better paradigm. The recruitment of a single synergy may increase the robustness and coherence levels across trials by reducing noise emerging from multiple synergies being recruited simultaneously. Furthermore, the scarcity of coherence does not unequivocally exclude cortical involvement, as coherence does not capture certain complex non-linear or cross-frequency interactions (Wang et al. 2014). Importantly, methods such as cross-frequency coupling are still evolving, and results derived from these methods should be interpreted with due caution (Aru et al. 2015). Another possible explanation for low coherence levels is subcortical influences replacing cortical involvement through motor development, maturity or training.

Delineating the functional communication across structures involved in the generation and modulation of muscle synergies, and discerning the variations based on weights within a synergy structure, is important for advancing our understanding of movement generation. In this study, we set out to identify muscle synergies that exhibit linearly synchronised neural activity. Our investigation had two primary aims: first, to identify the coherent activity across muscles of these identified synergies, and secondly, to examine the potential anatomical substrate correlated with these synergies by looking at dominant frequency bands. We postulated that an asymmetric distribution of a common efferent drive, implying subtle shifts in the synchronised neural activity to different muscles, could introduce adaptability within the synergy structure. With this purpose in minds, we examined a synergy-tuned task tailored to the preferred direction of each synergy.

## Materials and methods

### Participants

We recruited 13 right-handed volunteer participants (Table 1); participants did not present any pathology that affected the upper limb, spine or posture. Volunteers were excluded if they reported neck, shoulder, or arm pain (> 2 on a 1–10 verbal scale) within the last 3 months. The University of Auckland Human Participants Ethics Committee approved the research protocol and methods of the study (reference number 022246), and informed consent was gained before participation in any procedure.

**Table 1:** Participants characteristics

Variable	Age	Height (cm)	Weight (kg)	Arm Length (cm)	40% MVC (N)
Mean	26.5	173.4	72.3	65.4	31.0
SD	4.6	9.7	16.6	4.6	5.3

### Tasks and protocol

The participants attended a single session where they performed three upper limb isometric contractions tasks: maximal voluntary force (MVF), multidirectional trials exploring a high spatial volume, and synergy-tuned trials aimed at individual synergies’ preferred spatial directions. A similar protocol has been described previously (Ortega-Auriol et al. 2018).

The MVF tasks consisted of a maximum average force from three trials of external shoulder rotation. Shoulder’s external rotation is the weakest degree of freedom (DoF) for force development of the shoulder; consequently, forces in other DoFs will require less force relative to the MVF. Tasks involving low-to-moderate forces do not modulate coherence (Poston et al. 2010; Mima et al. 1999), but high force levels can shift the observed CMC from β to the lower γ band (Brown et al. 1998; Roh et al. 2012). A standardisation by shoulder external rotation allowed for low-to-moderate force levels across all directions.

**Fig. 1:**
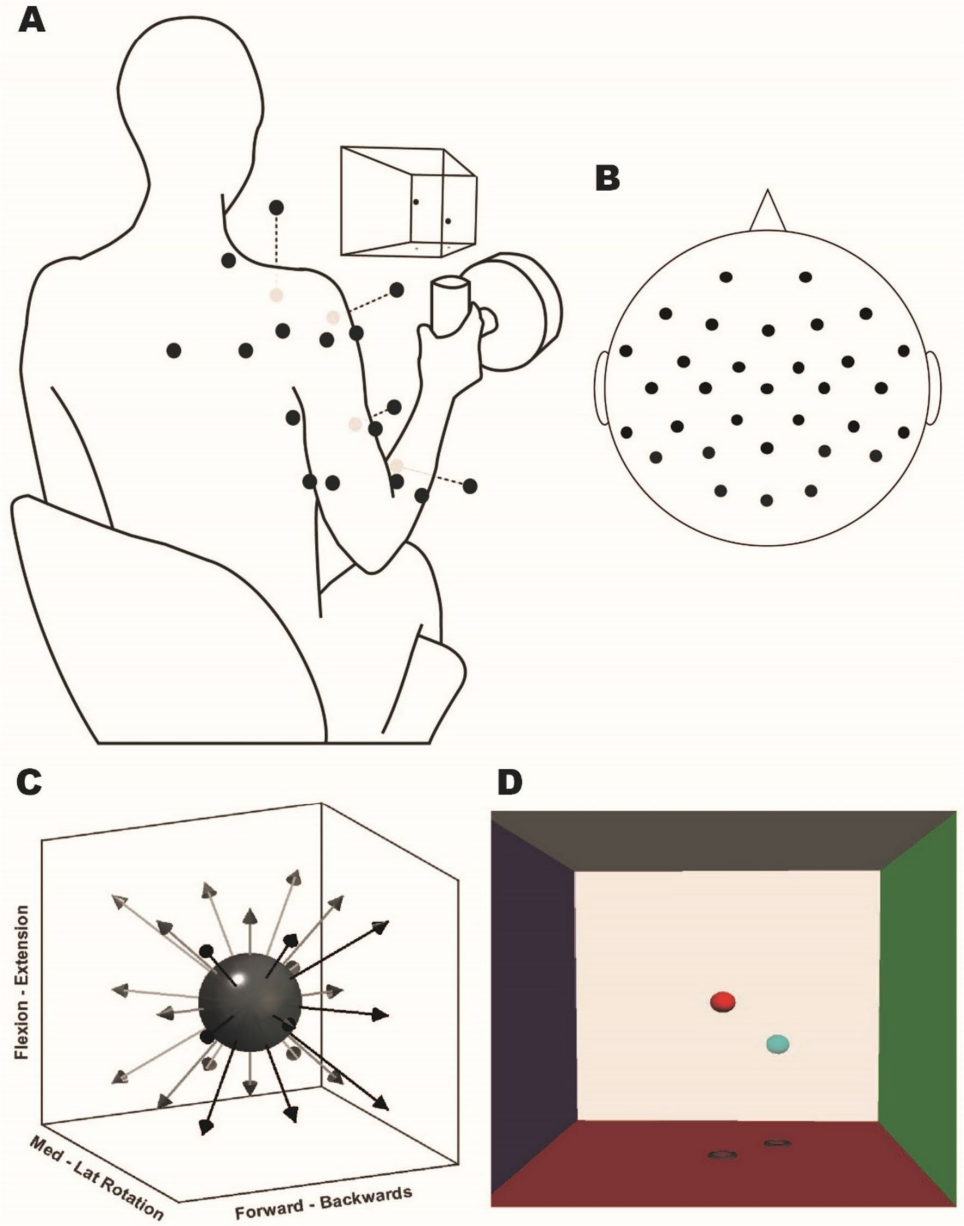
Experimental setup. **A** EMG sensor placement (black dots, grey dots are located ventrally), virtual reality feedback (VRF), and instrumented handle. **B** 10–20 EEG setup schematic. **C** Representation of target directions of the multidirectional task. **D** Screenshot of the VR feedback displayed on the screen. Each VRF wall is located 100 N away from the centre

During the multidirectional and synergy-tuned tasks, the participants were seated and exerted an isometric force with their dominant arm. The task trials were directed in specific spatial directions at 40% of the MVF using a handle instrumented with a force transducer (Fig. 1A). This force level was selected to ensure that participants could sustain the required isometric contraction in all directions without substantial effort, minimising the presence of fatigue and postural compensations. Trials were accepted if a 3D spatial target was achieved during four consecutive seconds within a resultant force range of ± 5 N. The handle was located at a position calculated as 40% of the arm length (acromion—3rd metacarpal head) in front of each participant’s shoulder, providing a comfortable position for participants to accomplish the desired protocol. The tasks consisted of matching a movable sphere to a target in a 3D visual representation, facilitated by real-time virtual reality feedback on a screen in front of them. This virtual reality feedback was complemented by shadows underneath the spheres, enhancing depth perception and guiding participants in reaching targets located in front or behind the initial sphere. Participants were given a training period prior to the tasks, allowing them to familiarise themselves with the task requirements and environment, ensuring a high level of performance and comfort during the actual trials. The distance vector between the spheres was 40% of the MVF, creating a clear and consistent objective for the participants during the task trials.

The multidirectional task consisted of isometric contractions to match targets in 26 different directions evenly distributed in a sphere (Fig. 1C). A significant number of muscle synergies were extracted from the multidirectional trials’ concatenated electromyography (EMG). From the activation coefficients of the extracted synergies, we determined the spatial tuning, known as the preferred direction (PD), of each of the extracted synergies. The synergy’s PD was determined as the direction in which the magnitude of activation coefficient of the synergy was maximal.

The synergy-tuned task consisted of several target matching trials towards the specific PD of a muscle synergy. The participants performed 50 trials in each synergy PD. The order of the trials was randomised and self-paced. The participants could rest between trials to avoid fatigue effects. A new synergy extraction was made from concatenated trials of each synergy’s PD. To corroborate the equivalency and conservations of synergies from the synergy-tuned trials, synergies were extracted again from the synergy-tuned task’s concatenated trials. Finally, CMC, CSC and IMC were calculated from the synergy-tuned trials.

### Recordings

Force was recorded at the handle with a 6-axis force transducer (Omega160, ATI Industrial Automation, USA) at 120 Hz. A Python-based custom software interface recorded signals. EEG signals were recorded from a 32 Ag/AgCl electrode EEG system (EasyCap; Brain Products GmbH, Germany). The electrodes were positioned according to the 10–20 system, referenced to the FCz channel, and offline to a common reference. Signals were acquired with BrainVision Recorder software (Brain Products GmbH, Germany) at 5 kHz.

Surface EMG signals were recorded from 16 single differential channels and sampled at 2 kHz using a Trigno device (Delsys Inc., USA). EMG activity was recorded from muscles of the participant’s dominant upper limb: superior (ST) and middle trapezius (MT), infraspinatus (Inf), teres minor (TM), serratus anterior (SA), anterior (AD), middle (MD), and posterior deltoid (PDel), pectoralis major (PM, clavicular fibres), short (BS) and long (BL) heads of biceps brachii, long (TL) and lateral (Tlat) heads of triceps brachii, brachioradialis (Braq), extensor carpi radialis (ECR), and flexor carpi radialis (FCR). These muscles were chosen based on their force capability and likely contribution to the required task, as essential considerations for accurate reconstruction of synergies (Steele et al. 2013). Electrodes were positioned according to SENIAM and Cram’s guidelines (Hermens et al. 1999; Criswell 2010). The participant’s skin was prepared with a gentle abrasive gel to clean and improve transmission before placing the electrodes.

**Fig. 2:**
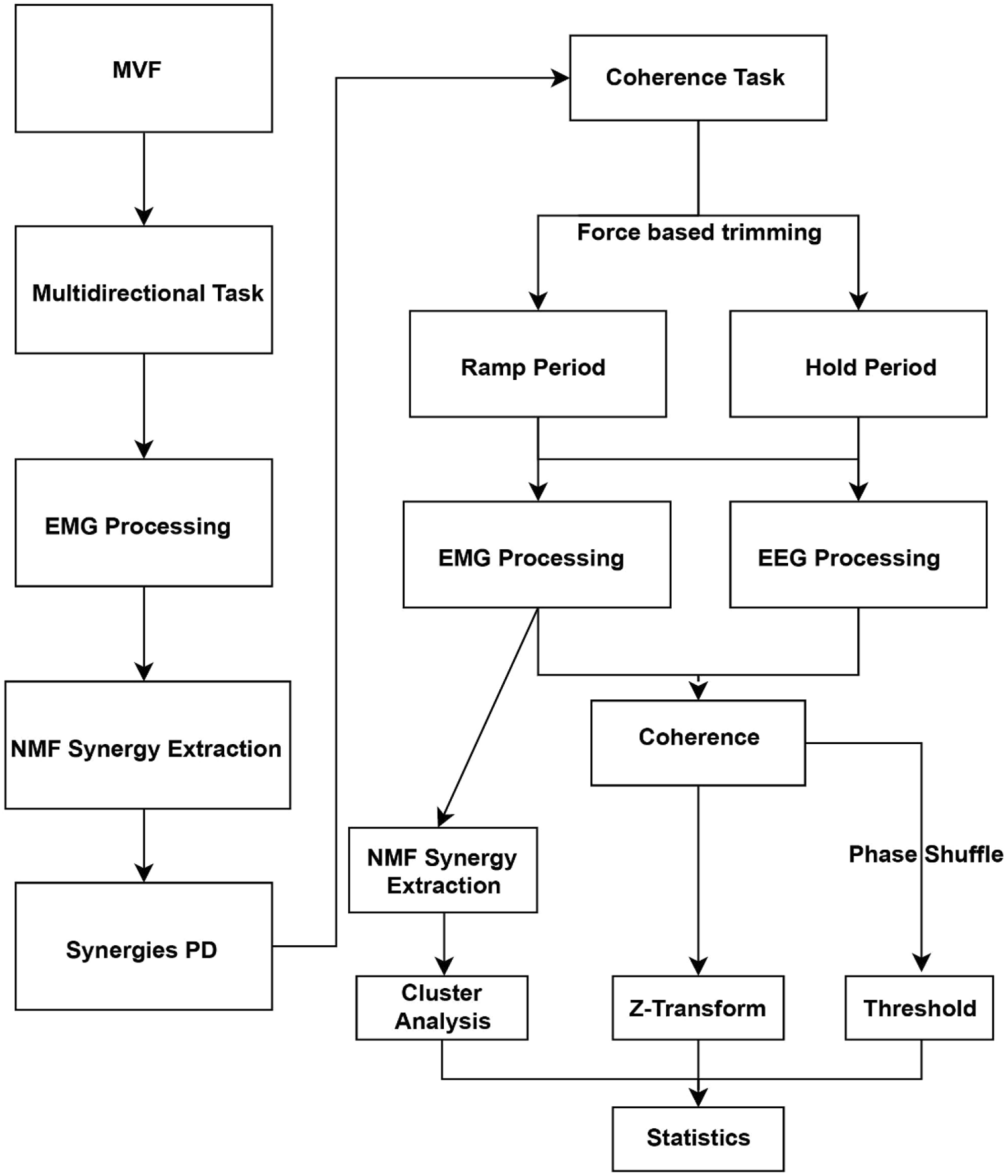
Data processing pipeline from MVF to statistical analysis

Synchronisation across devices, EEG, EMG, and force acquisition were performed with Python-based custom software (https://dragonflymessaging.org/applications.html, U. of Pittsburgh). Data analysis was performed in MATLAB 9.3 (MathWorks, USA) using custom-made scripts and the FieldTrip toolbox (Oostenveld et al. 2011). A schematic representation of the workflow to process the data is shown in Fig. 2.

### Data analysis

**Fig. 3:**
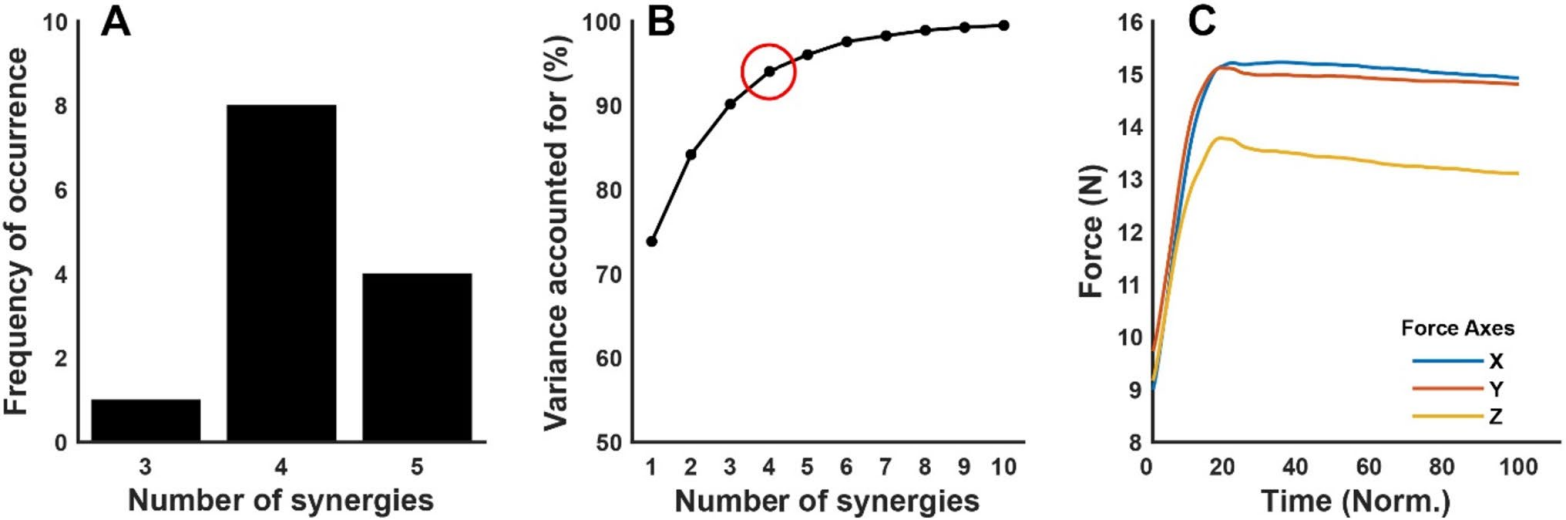
Multidirectional task results. **A** Frequency of occurrence of extracted synergies per participant. Since five was the highest number of synergies across participants, the same number of clusters were extracted for subsequent analyses. **B** Mean VAF across participants for the extraction of N synergies, the synergies’ mode is noted with a red circle. **C** Average force traces across all participants and trials

To process the EMG and EEG signals, all trials were split into a ramp and a hold phase based on the force profile. The ramp phase of a trial was defined as the time window from the initial movement of the feedback sphere until the force trace’s inflexion point (knee). A trial’s hold phase was defined as the intermediate 2 s of the required 4 s of target matching of a multidirectional or synergy-tuned trial. To trim the trials, force data (Fig. 3) were low-pass filtered (Butterworth, second-order, 5 Hz), and the inflexion point of the force traces was calculated by a custom algorithm and corrected through visual inspection if necessary.

#### EMG

The pre-processing of EMG signals for synergy extraction is described in detail in a previous report (Ortega-Auriol et al. 2018). Briefly, EMG signals were: band-pass filtered (bidirectional Butterworth, 2nd order, 5–400 Hz), demeaned, full-wave rectified, normalised to maximum activation across trials for each muscle to preserve relative contribution, converted to unit variance, low-pass filtered again to obtain an envelope (Butterworth, 2nd order, 5 Hz), and, only for synergy extraction, rebinned into 100 data points.

#### Synergy extraction

Non-negative matrix factorisation (Lee and Seung 1999) was applied individually to the processed concatenated EMG signals from the multidirectional and synergy-tuned tasks. NMF can be modelled as *D* = *W* × *C *+ *ϵ*, where *D* is the original data set, *W* the synergy structure and *C* the activation coefficients, and *ϵ* is the variance not explained by the synergies. NMF was implemented using the multiplicative rule (Berry et al. 2007). The final solution was the resultant of 20 consecutive iterations with a difference of EMG reconstruction error smaller than 0.01% among them. First, to determine a significant number of synergies, the algorithm iterated from one until the number of muscles minus one. Second, we used the VAF metric (Cheung et al. 2005) to determine the number of synergies that achieved the best reconstruction of the original data. VAF was applied as a global (quality of original dataset reconstruction) and local criterion (quality of individual muscles’ signal reconstruction). A significant number of synergies was determined when global VAF ≥ 90% and local VAF ≥ 80% (Cheung et al. 2005; Chvatal and Ting 2012; Kim et al. 2016; Ortega-Auriol et al. 2018).

#### Synergy preferred directions

Once a significant number of synergies were determined from the multidirectional task, the PD of each synergy was computed as the average of each trial’s target direction scaled by the activation coefficient of that synergy during that trial (Eq. 1):

1 $$\overset{\lower0.5em\hbox{$\smash{\rightharpoonup}$}}{{{\text{PD}}_{r} }} = \frac{{\sum i(Q_{i} \times C_{ri} )}}{T},$$

where *Q*_*i*_ is the direction unit vector of the *i*th trial, *C*_*ri*_ is the activation coefficient of the *r*th synergy of the *i*th trial, and *T* is the total number of trials. In a previous publication (Ortega-Auriol et al. 2018), our results showed that synergies’ activation coefficients are directionally scaled in the explored spatial volume, allowing the identification of synergy PDs.

#### Synergy-tuned trial pre-processing

EEG and EMG data from the synergy-tuned task were band-pass filtered (bidirectional Butterworth, second-order, 5–400 Hz), demeaned, EMG data were rectified via Hilbert Transform, and each trial was split into a ramp and a hold phase. Signals were rectified to emphasise the grouping and timing of action potentials within the EMG signals, as recommended for correlation and coherence analysis (Boonstra and Breakspear 2011; Farina et al. 2013; Ward et al. 2013). EEG signal impedance was checked at two instances during preparation: before starting data collection and in the time window between the multidirectional and synergy-tuned tasks. Electrodes with impedance levels over 15 Ohms were adjusted. EEG was downsampled to 2 kHz to match the EMG sampling frequency and optimise processing times. Independent Component Analysis was applied to EEG to remove electro-oculographic artefacts. To remove electro-oculographic artefacts while conserving the integrity of the data, a single component which visually presented artefacts and was located in the frontal region, was subtracted from the EEG data reconstruction.

#### Cluster analysis

To group similar synergies across participants, a cluster analysis was applied (García-Cossio et al. 2014; Roh et al. 2015) to the pooled synergy structures of all participants from the multidirectional and synergy-tuned tasks. Cluster analysis was applied using a k-medoids algorithm (Park and Jun 2009) with a cosine function as the cluster distance metric between members and the cluster’s centroids. The number of clusters was fixed to the maximum number of extracted synergies across participants. Membership assignment within a cluster was constrained to prevent the inclusion of two or more synergies from a single participant within a cluster. In these cases, the closest synergy to the centroid of the next nearest available cluster was reassigned. The reassignment process was iterated until no further repetitions were found. The synergy grouping results across participants is displayed as a mean synergy for each cluster.

#### Coherence calculation

IMC, CMC and CSC were calculated from the concatenated trials from each set of synergy-tuned trials in a single direction. To confirm consistency between the extracted synergies from the synergy-tuned trials and those from the multidirectional task, we compared them using cross-correlation and dot product. The synergies’ structures from the synergy-tuned task were not different from those extracted from the multidirectional task. IMC was calculated between three different muscle groups: (A) all–all, representing the average IMC of all muscles pairs within a single synergy, (B) high–high, being the highest IMC across the three muscles with the highest weights within a synergy, and (C) high–low displaying the highest IMC across three highest and the lowest weight muscles within each synergy. Muscle selection for group comparison was based on the muscles’ weights within a synergy. Muscle weights were determined in two different ways, first based on the mean synergies across participants and secondly on the individual synergy structure of each participant. CMC was calculated between each EMG channel and EEG channel in the motor area. CSC was calculated between the activation coefficients of the single synergy extracted per set of synergy-tuned trials and the respective EEG data.

After pre-processing, EMG, synergies’ activation coefficients, and EEG signals were transformed into the frequency domain to calculate the coherence measures. A fast Fourier Transformation (FFT) was applied to the bandwidth between 3 and 50 Hz. FFT results consisted of 24 frequency bins between 3 and 50 Hz with steps of 2 Hz. To narrow the scope of CMC and CSC calculations, we only considered channels on and near the motor cortex area: FC5, FC1, Fz, Cz, C3, T7, CP5, and CP1. CMC and IMC were calculated for the available combinations between EEG–EMG and EMG–EMG channels. CSC was calculated between a single activation coefficient and a set of synergy-tuned trials of EEG data. From the subset of analysed EEG channels, the one with the highest average CMC or CSC value was used for further analysis. Raw coherence (Rosenberg et al. 1989) was calculated using the FieldTrip toolbox (Oostenveld et al. 2011) applying Eq. (2):

2 $$C_{xy} (f) = \frac{{|P_{xy} (f)|^{2} }}{{||P_{xx} (f)P_{yy} (f)||}},$$

and values were normalised by applying a z-transformation (Baker et al. 2003; Reyes et al. 2017) using Eq. (3):

3 $$Z = \frac{{\arctan h\left( {\sqrt c } \right)}}{{\sqrt {1/2N} }},$$

where *c* is the raw coherence value, and *N* is the number of tapers, which was four, used in the coherence calculation. From the individual estimates of coherence, pooled CMC, CSC and IMC were calculated to produce a single global estimate of correlated CMC or IMC (Amjad et al. 1997).

A significance threshold based on a surrogate coherence analysis derived from the original EMG and EEG data was calculated to define a significance level for the coherence calculations. Once the original data were transformed into the frequency domain, the argument of the complex quantity (angle of the polar form) was independently shuffled. The shuffling was iterated 50 times across all trials, channels, and participants. Then, coherence was calculated as described previously. This procedure allows the conservation of the power spectrum original amplitude structure of the signal while only shifting the signal phase, uncorrelating the signals in the time and frequency domain (Faes et al. 2004; Marchis et al. 2015). Coherence threshold significance was established as above the 90th percentile of the resultant by-chance coherence distribution.

**Fig. 4:**
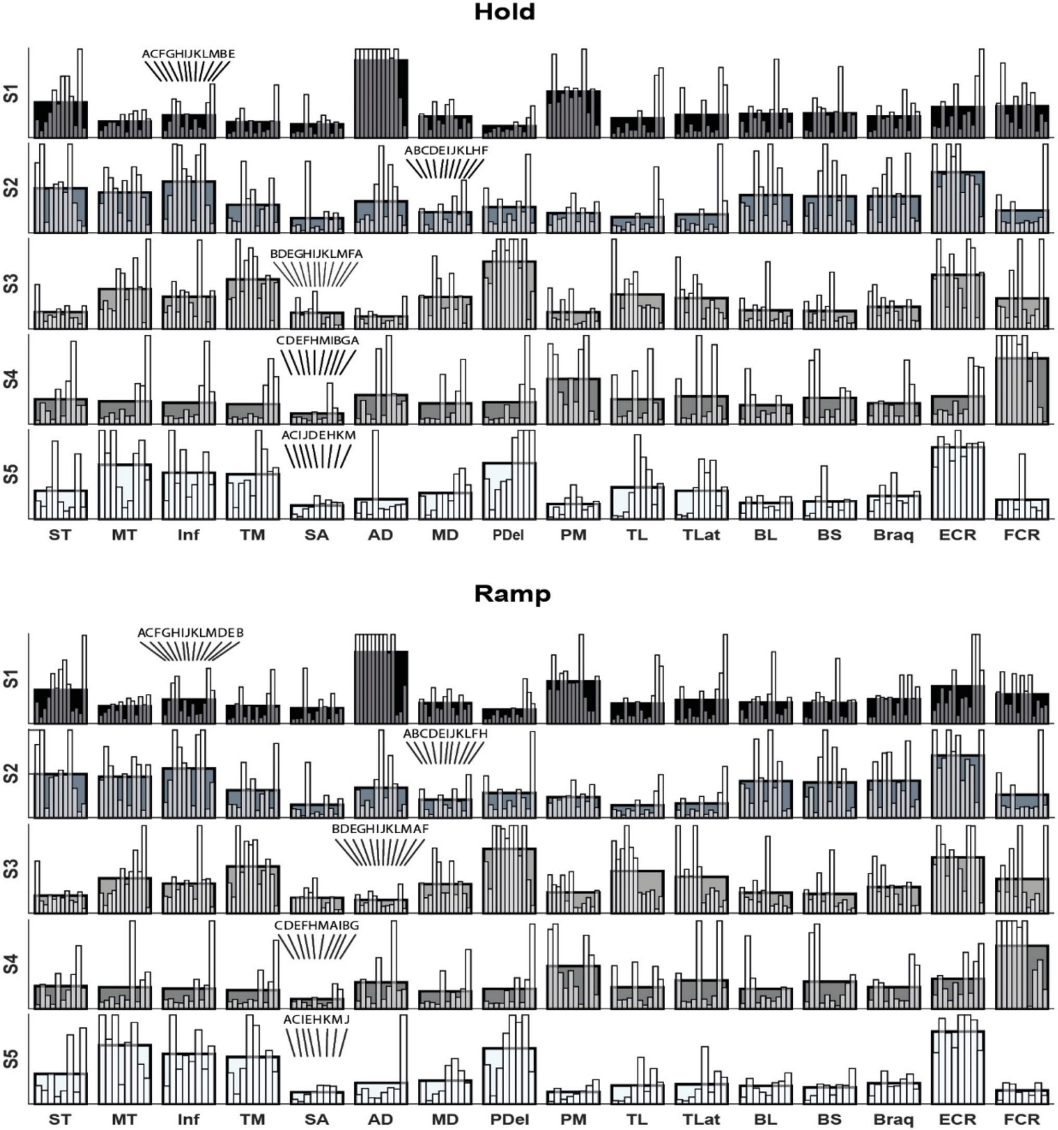
Individual (white overlaid bars) and cluster means of normalised synergies (greyscale bars) for ramp and hold phases. Five different clusters (S1–S5) of synergies were calculated according to the maximum number of identified synergies across participants. Letters above the white overlaid bars correspond to each participant, as in Table 1. Muscles are labelled in an abbreviated form: superior (ST) and middle trapezius (MT), infraspinatus (Inf), teres minor (TM), serratus anterior (SA), anterior (AD), middle (MD), and posterior deltoid (PDel), pectoralis major (PM), short (BS) and long (BL) heads of biceps brachii, long (TL) and lateral (Tlat) heads of triceps brachii, brachioradialis (Braq), extensor carpi radialis (ECR), and flexor carpi radialis (FCR)

**Fig. 5:**
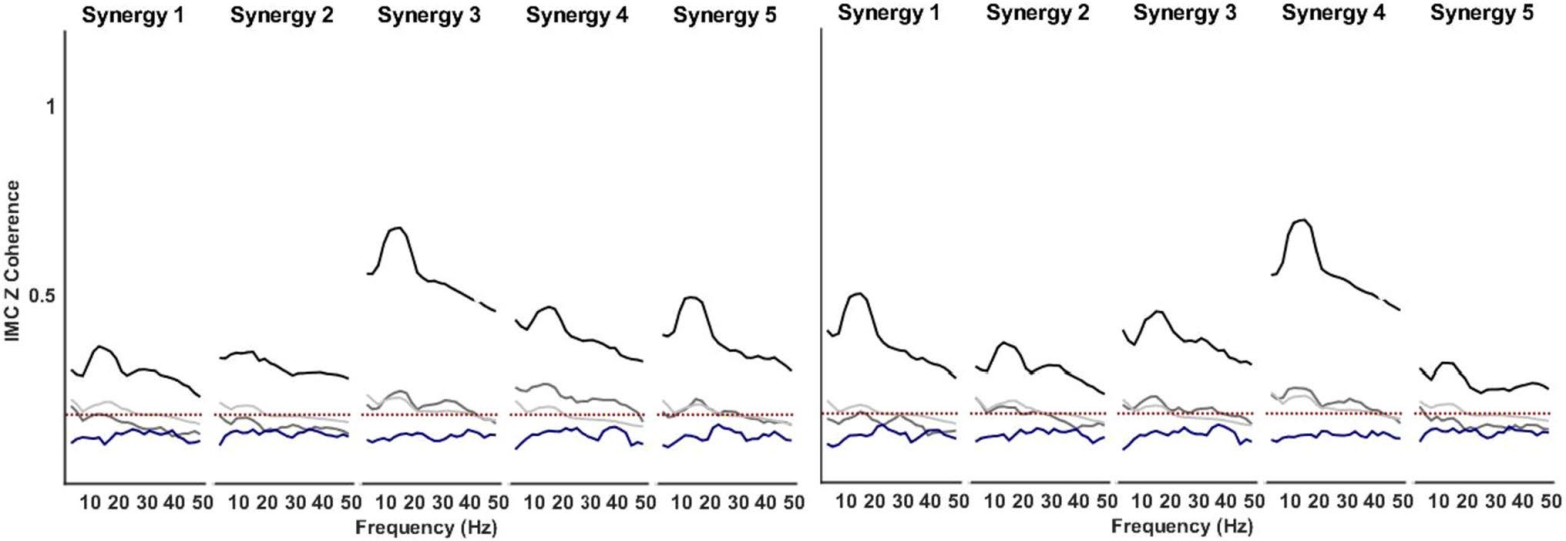
Ramp (left) and hold (right) phases cluster averages of CMC (navy trace) and IMC for three different groupings: all–all (silver trace), high–low (grey trace) and high–high (black trace) and threshold (red dotted trace)

Data distributions were checked for normality using a Kolmogorov–Smirnov Test (*k*-test), assessing for skewness and visually inspecting a normality plot. Then we compared the average IMC between different muscle groups within each synergy (high–all, high–high, and high–low, Fig. 5) using Friedman’s ANOVA; this analysis was constrained within the most relevant frequency bands (7–20 Hz, Fig. 4) around the observed peak.

## Results

All participants (Table 1) completed the multidirectional and synergy-tuned trials on the requested directions and number of repetitions. On average, from multidirectional trials, 4.2 (SD 0.6) synergies were sufficient to reconstruct the original EMG data set (VAF = 94.2, SD 1.4). On average, MVF was 77.6 N (SD 12.2), requiring 31 N (SD 5.3) as 40% of the MVF (Fig. 3).

### Muscle synergies

Five synergy clusters were calculated from the pooled synergies from the multidirectional task across participants (Fig. 4). S1 is a shoulder flexion/internal rotation synergy with contributions from the AD and PM muscles. S2 is a shoulder external rotation and elbow flexion synergy (an outward pulling action), with the contribution of the MT and ST as scapula stabilisers while the Inf muscle contributes to the rotation. S3 can be interpreted as a synergy for shoulder extension with external rotation and elbow extensor, involving the PDel, TM, and contribution from triceps muscles. S4 performs shoulder adduction and elbow extension with internal rotation, with high weights for FCR and PM. Finally, S5 is a shoulder–elbow extension synergy (a downward pulling action) involving the PDel scapula stabilisers and triceps muscle. This functional interpretation resembles an orthogonal distribution of the extracted synergies crossing the shoulder joint.

### CMC and IMC

We first calculated CMC and IMC individually for each participant from every direction of the synergy-tuned trial. The higher weight muscles per cluster were: S1 = [AD, PM, PDel], S2 = [Inf, ST, TL], S3 = [PDel, TM, MD], S4 = [PM, AD, BL], and S5 = [PDel, Inf, PM]. CMC at all frequencies, calculated as the average of all muscle pairs (all–all), was below the threshold during both the ramp and hold phases. Conversely, IMC of all muscle pairs (all–all) was predominantly on or just above the threshold. The high–high group showed coherent activity in all five synergy clusters for most participants (66%) across both phases (Fig. 5).

### Coherence and synergy weight interaction

**Fig. 6:**
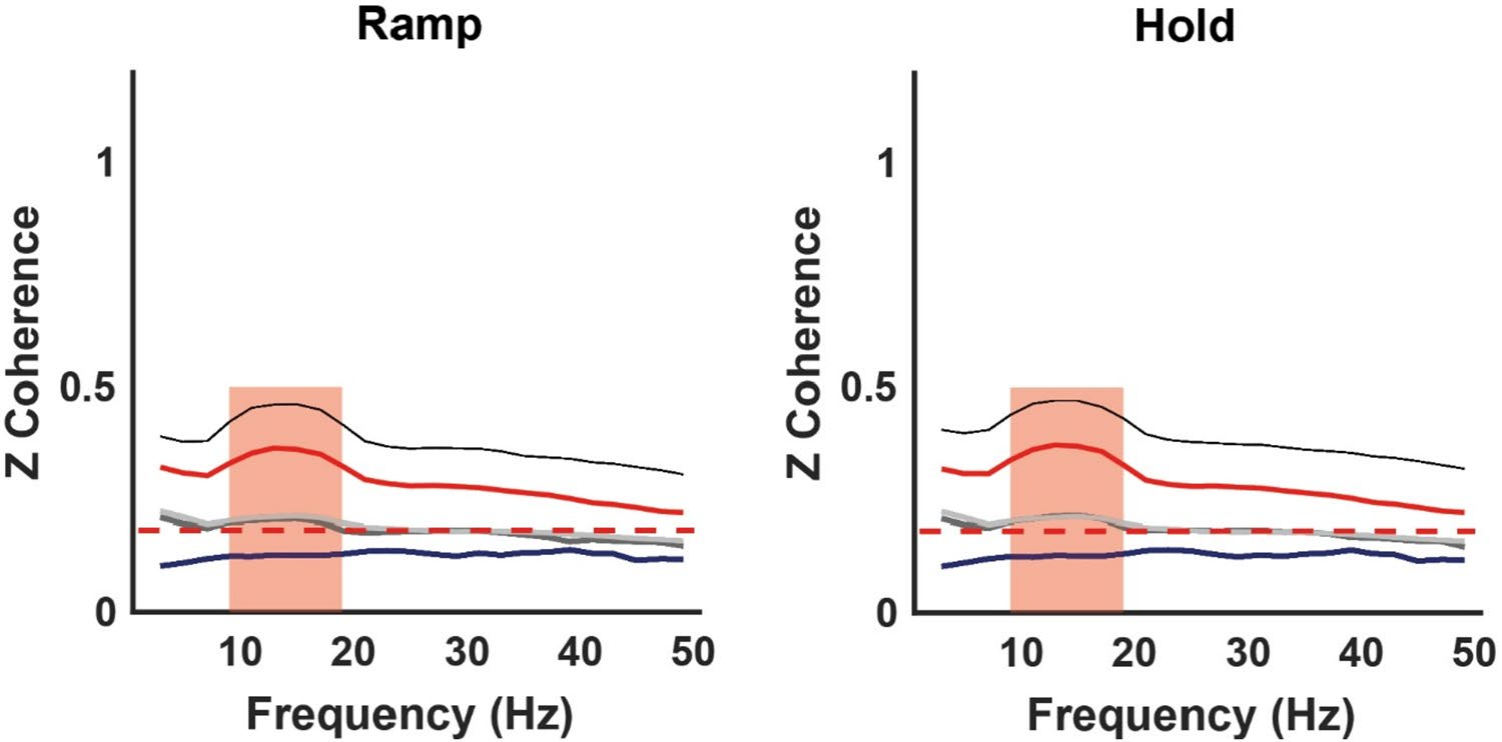
Ramp (left) and hold (right) phases average across synergies of CMC (navy trace), IMC on four different groupings: all–all (silver trace), high–low (grey trace) and high–high by individual contributor selection (black trace) and by mean synergy contributor selection (red trace), and threshold (red dotted trace). The red patch highlights the relevant frequencies (7–19 Hz)

**Fig. 7:**
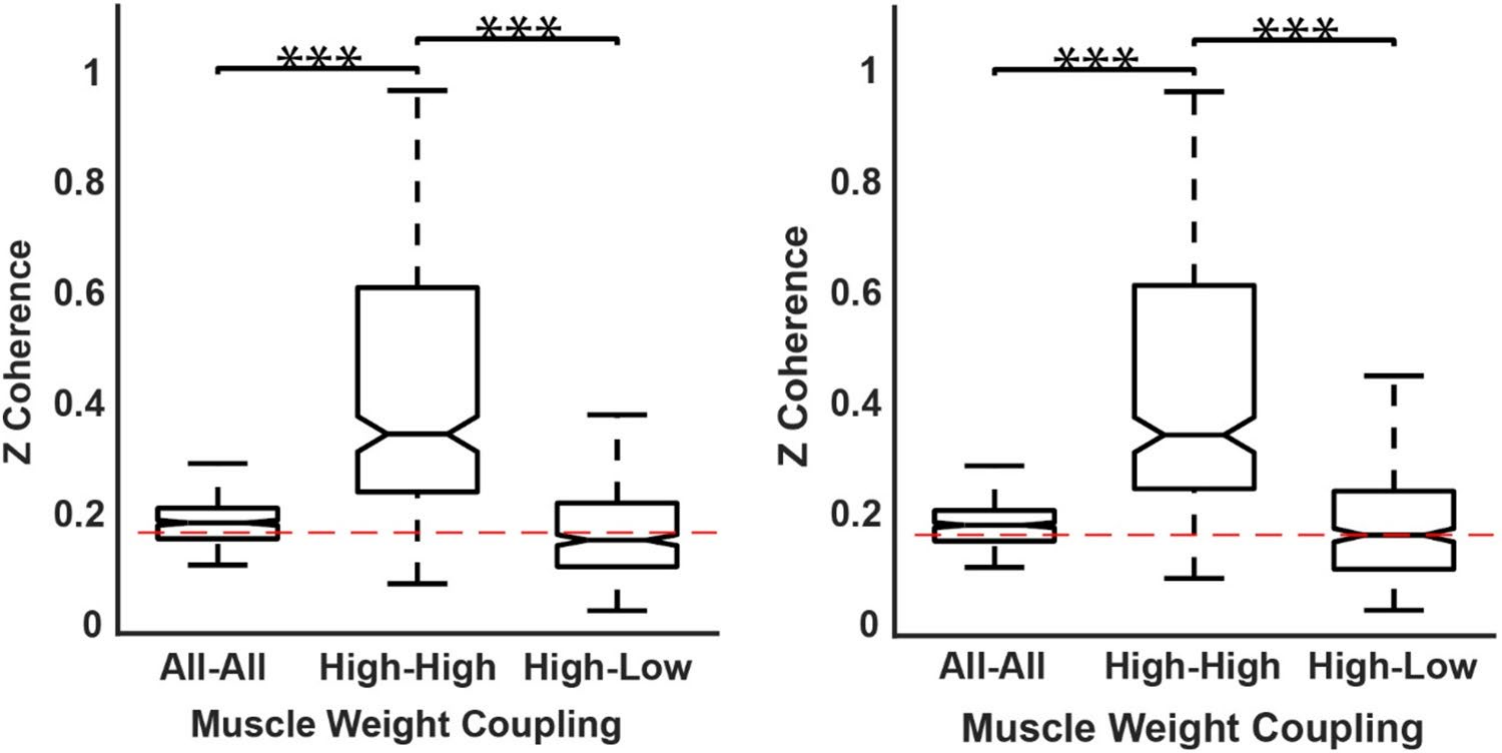
Statistical comparison of the IMC levels for each muscle grouping, averaged across all frequencies, during the ramp (left) and the hold (right) task phases

Comparisons of IMC levels between the pairing groups were made around the peak frequency (7–19 Hz, Fig. 6, red patch; Fig. 7). IMC of the high–high group was not normally distributed [*k*-test *p* = 0.0002, skewness = 0.5]. Similarly, the high–low [*k*-test *p* = 0.002, skewness = 0.8] distributions were significantly non-normal. Therefore, we checked the mean difference between the IMC distributions by applying Friedman’s ANOVA. The IMC level was different across the three different IMC groups for the hold ($x^{2} (2) = 97.0,\;\;p = 0.008$) and ramp ($x^{2} (2) = 75.7,\;\;p = 0.007$) phases. Wilcoxon post hoc tests were used to find individual group differences. For the hold phase, the high–high IMC was significantly different from high–low ($z = 8.3,\;\;p = 0.0006$) with a large effect size (ES) = 0.81 (Rosenthal 1986) and different from the average of all muscles IMC ($z = 7.8,\;\;p = 0.0003$) with a medium ES = 0.76; no significant difference was found between high–all and high–low IMC. Similarly, for the ramp phase, the high–high IMC was significantly different from high–low ($z = 8.1,\;\;p = 0.0005$) with a medium ES = 0.78 and different from all–all IMC ($z = 7.5,\;\;p = 0.0003$) with a medium ES = 0.72, and no significant difference was found between high–all and high–low IMC. Interestingly, even though synergies within a cluster are fairly consistent, when muscles were selected based on the cluster mean synergy structure rather than on an individual basis, the overall IMC level was lower (Fig. 6).

### Cortico-synergy coherence

**Fig. 8:**
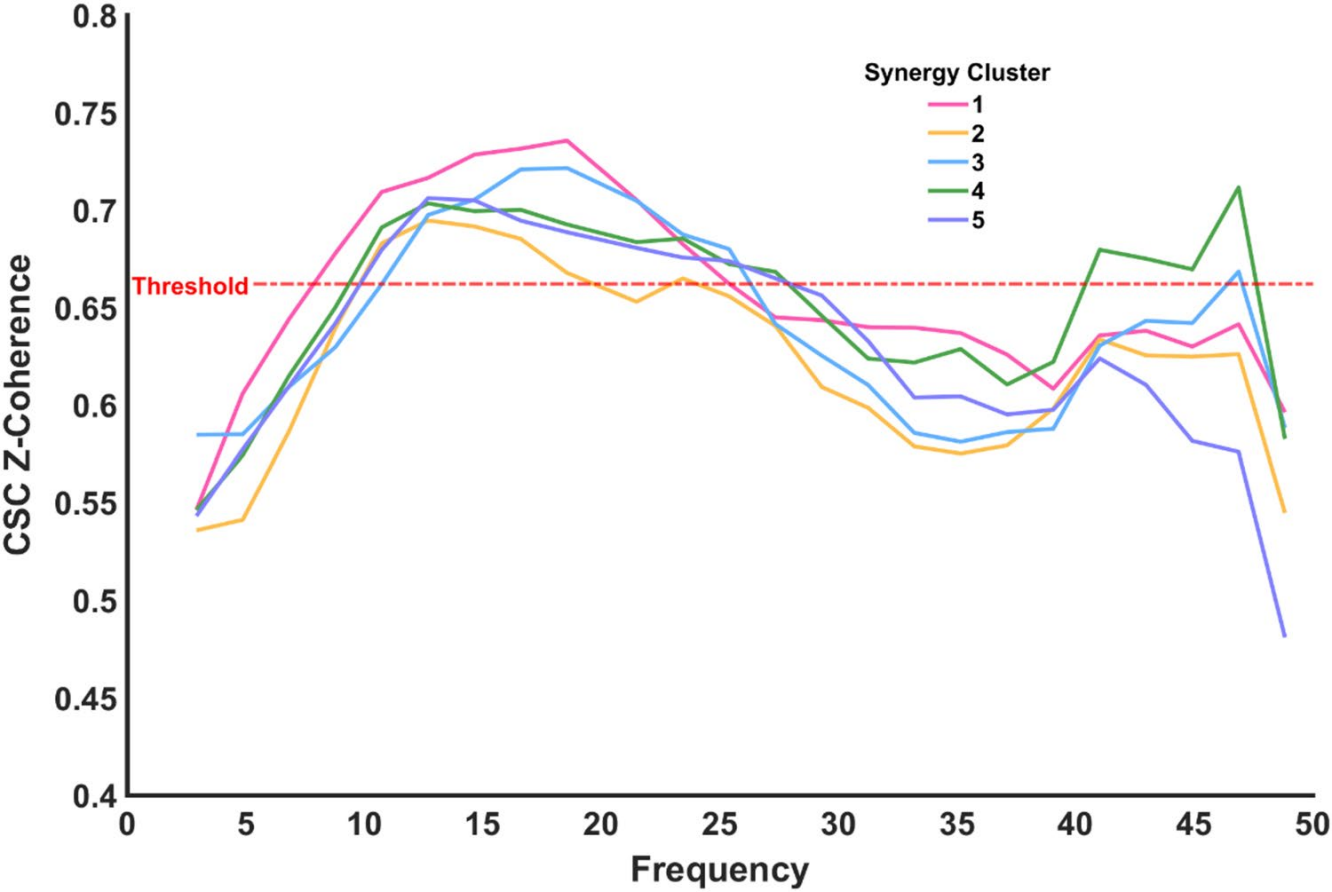
Cortico-synergy coherence of all synergy clusters and significance threshold. CSC was calculated between the activation coefficient of the members of a cluster and the EEG activity of the electrodes on the motor cortex area

To further test cortical involvement in synergy development of the upper limb, we calculated CSC (Fig. 8). CSC was significant for all synergies around the α and β frequencies. In addition, a second increase of CSC was present in the gamma band, although this was significant only for the 4th synergy cluster.

Data can be made available from the corresponding author upon reasonable request.

## Discussion

To elucidate the functional connectivity underlying muscle synergy formation during isometric arm tasks EEG-EMG, EMG-EMG and Synergy-EEG coherence were examined in this study. Three key results were found. First, a significant level of IMC in the α and β frequencies for each synergy. Synergy structure influenced the IMC levels, where higher weight muscles showed higher levels of IMC. Second, a significant CSC level for all synergies in the β band and a significant level for a single synergy in the γ band. Third, the absence of CMC during the synergy-tuned task. These findings highlight the correlated activity between muscles, the synergies weighted structure, and between synergies and brain activity, thus highlighting a neural strategy for simplified motor control. Intermuscular coherence (IMC) in the alpha and beta frequencies, particularly pronounced in higher weight muscles, suggests that synergy structure dictates the neural coordination of muscles. Cortico-synergy coherence (CSC) in the beta band, and for a single synergy in the gamma band, strengthens the idea of the motor cortex's involvement in synergy generation and control. The absence of cortico-muscular coherence (CMC) during the synergy-tuned task suggests a more distributed, synergistic control of movement at the cortical level, rather than direct control of individual muscles. These results are relevant to the understanding of the mechanisms of muscle synergies within the broader context of modularity in motor control.

### Muscle contributions within a synergy

The results showed higher IMC levels between two muscles of high contribution (high–high) within an active synergy than between muscles with high–low contributions within the same synergy. From a functional perspective, muscles with a higher contribution within a synergy have a role as the primary movers when performing motor tasks in the synergy’s PD. High coherence levels suggest either a shared excitatory drive (Danna-Dos-Santos et al. 2014) or a closed-loop network (Aumann and Prut 2015). Our results align with the idea that functionally or anatomically related muscles share a common input (Farmer et al. 1993; Gibbs et al. 1995; Kerkman et al. 2018). Similarly, studies have proposed that the motor control system closely supervises primary movers in a motor task (Krishnamoorthy et al. 2003; Danna-Dos-Santos et al. 2007). The study findings were consistent across all the extracted clusters and are consistent with the modules found across whole body functional and anatomical networks (Boonstra et al. 2015; Kerkman et al. 2018). Therefore, our data are consistent with a long-range synchronisation by high IMC level and even higher values in those muscles acting as the primary movers that would require closer supervision. The implication of a shared neural drive or network with closer supervision of the main contributors within each synergy is that our results suggest synergies as a hard-wired neurophysiological control strategy.

The difference in IMC between high–high and high–low muscle pairs was more pronounced when the dominant muscles were selected based on an individual’s synergy structure basis rather than the clustered synergies’ average structure. This result highlights that even though the cluster average structures resembled the reconstructed synergies from each individual, the differences between cluster average and individual structures were significant with respect to their functional connectivity, independent of the anatomical structure. Genetic (Goodman and Shatz 1993) and developmental (Ritterband-Rosenbaum et al. 2017) factors could lead to individual differences in the anatomy and neurophysiology of each individual. The conserved sensitivity of IMC to an individual’s synergy structure reflects a common principle of neuromuscular control that overarches any individual differences that result from these developmental influences.

Previous research (Marchis et al. 2015) found that only one task-specific synergy out of four identified synergies had a significant IMC level in the γ band between high-weight muscles during dynamic pedalling conditions; and γ band activity suggests a common cortical input under dynamic conditions (Omlor et al. 2007). Our results, on the other hand, have shown consistent IMC in the α and β bands in every synergy cluster, even across topographically distant muscles. This suggests neurological underpinnings, as IMC between distant muscles cannot be explained by afferent systems, sensory environments, or mechanical demands alone. In addition, α band IMC emerges during a sustained low-to-moderate force exertion paradigm (Boonstra 2009) that closely matches our experimental task. However, IMC is sensitive to experimental conditions, which may partially explain differences between our results and those of other studies (Danna-Dos-Santos et al. 2014; Marchis et al. 2015). To improve the identification of synergies’ underlying neural mechanisms, we used synergy-tuned target directions to optimise the recruitment of a single synergy and reduce the ‘noise’ from multiple synergies being recruited simultaneously.

### Cortico-synergy coherence

Each frequency band component of signals has been suggested to have a specific role in motor behaviour (McAuley and Marsden 2000). Studies have suggested low frequency components (0–3 Hz) reflect afferent pathways, α components represent subcortical involvement (Boonstra 2009; Marchis et al. 2015), β band corticospinal projections (Conway et al. 1995; Baker et al. 2003), and higher components (> 30 Hz) may reflect propriospinal pathways (Levine et al. 2014; Kerkman et al. 2018).

Our results indicate above threshold α CSC, a frequency band that has been associated with motor correction, locomotion and reaching. The α band is typically considered to be of subcortical origin, given its lack of linear synchronisation with cortical activity (Conway et al. 1995; Brown et al. 1998; Baker et al. 2003). Evidence suggests that α band activity is linked to the involvement of the reticulospinal pathway, which is associated to locomotion control (Matsuyama and Drew 2000), postural adjustments and reaching (Schepens and Drew 2004; Buford and Davidson 2004). Interestingly reaching movements and isometric contractions of the upper limb share a large common base of synergies (Pham et al. 2023). Therefore, the presence of correlated activity within the α band may provide a plausible mechanism for executing and adjusting upper limb isometric contractions. Existing evidence, concurrent with our study, also highlights the modulation of muscle synergies by the α band, illustrating its potential role in the coordination of motor functions (Laine et al. 2021).Our results also showed high levels of β IMC and CSC, which have previously been found during isometric motor tasks of the upper limbs (Conway et al. 1995; Kilner et al. 1999; Baker et al. 2003; Aumann and Fetz 2004). Moreover, β-band synchronous activity has also been associated with muscle synergies (Aumann and Prut 2015; Zandvoort et al. 2019). Evidence also suggests the possible cortical representation of synergies within the motor cortex (Schieber 2001). The resonance of β activity along the central and peripheral neuromusculoskeletal system indicates the presence of a long-range synchronisation network with cortical representation and modulation. While it is impossible to determine the nature of the network, given that coherence is a non-directional method, it is possible that the network is either in a closed-loop or derived from a common oscillator (Aumann and Prut 2015). A higher synchronisation between a cortical efferent with synergies’ activation coefficients and between high-weight muscles within a muscle synergy suggests a closed-loop network, allowing differential loops for individual synergies, providing the necessary flexibility to merge them and produce complex movements (Nazarpour et al. 2012; Aumann and Prut 2015).

### Cortico-muscular coherence

Several factors associated with the experimental motor task may explain the lack of CMC in our results. First, variable force outputs decrease CMC, which can transfer to subsequent periods of constant force production (Kilner et al. 1999; Boonstra 2009). This CMC suppression could be manifest within the task considering the ramp phase as a variable force output (Fig. 3C). Second, reductions in CMC have been associated with increases in IMC due to coordinated functional activity between muscles (de Vries et al. 2016). Thus, our task resulting in high β band IMC levels due to the recruitment and the co-activation between muscles of the UL (Lee et al. 2014) would again result in a low CMC level. Third, while precise grips involving both intrinsic and extrinsic muscles of the hand have been found to increase CMC (Kilner et al. 1999, 2000; Boonstra 2009), our experiment involves low hand precision. Low precision has been linked with decreased CMC levels (Kristeva-Feige et al. 2002). Fourth, the performance of a cognitive task while executing a motor task also reduces CMC (Kristeva-Feige et al. 2002). Our task required a certain level of cognitive involvement from the 3D spatial nature of the force target; participants had to pay attention to several details on the screen to match the target. Finally, CMC does not reach significant levels during low-to-moderate contractions (Mima et al. 1999; Poston et al. 2010). On the contrary, high force levels could modulate CMC from a beta to a low gamma rhythm (Brown et al. 1998). Our task required a force target of 40% of the external shoulder rotation MVF, being the weakest force across the shoulder degrees of freedom. Thus, the forces generated in all other directions required low-to-moderate forces relative to their own MVF which on average could mask any significant levels of CMC.

Another explanation for the lack of CMC could arise from developmental factors. While the basic structure of some synergies, encoded on well-defined spinal cord networks, remains unchanged from early childhood, their modulation may vary as motor maturity is reached (Dominici et al. 2011). Particularly for proximal upper limb muscles during isometric tasks, the necessity for direct CMC might reduce, as these tasks could be largely governed by the established spinal circuits, but their recruitment still be modulated by CSC. By the time motor maturity is reached in adolescence more synergies have developed, allowing the production of complex movements (Dominici et al. 2011). Our task consists of a relatively simple motor behaviour, making it possible that only the more ‘primitive’ synergies encoded in the spine are necessary to perform the task. Despite their unchanged structure, the modulation of these synergies could primarily serve for fine-tuning and adjustment of movements rather than direct task execution. As reflected by the presence of CSC, CMC could remain low. Therefore, further investigation into the task constraints which permit CMC is warranted.

Identifying the spatial tuning of muscle synergies during specific tasks can provide valuable insights for tailored rehabilitation strategies. Such an approach would allow therapists to isolate and target individual synergies, thereby improving the specificity and efficiency of the rehabilitation process. This paradigm of targeted synergy recruitment can be implemented initially in isolation, and later combined to address more complex motor behaviours. Given that the preferred direction of recruitment can vary among individuals, this could further enable personalised therapeutic interventions.

In addition, evidence suggests that both isometric tasks and dynamic reaching movements recruit some common muscle synergies (Pham et al. 2023), despite each having unique synergies also. Therefore, improvements in coordination achieved through isometric training could potentially be transferable to dynamic behaviours. It is also relevant to mention the correlation between coherence levels and paresis, even when the motor tracts seem intact, therefore, highlighting the importance of functional connectivity with movement quality. These concepts underline the viability of employing isometric task paradigms in rehabilitation settings for enhancing coordination across a spectrum of motor activities. However, the extent of this transferability requires further investigation. This approach to understanding and manipulating muscle synergies presents a possibility for developing more effective and individualised neurorehabilitation strategies.

## Limitations

Several methodological and statistical considerations and limitations must be considered for a correct interpretation of our results. First, signal filtering influences the number of synergies extracted (Santuz et al. 2016). When choosing the correct signal-to-noise ratio level, there is no clear cutoff point, but over-filtering could potentially remove significant motor variability from the signals. However, any bias introduced by filtering would be consistent across participants, thus affecting coherence calculations equally across the results. Second, to conserve a one-to-one relationship between a synergy and its PD, we constrained the synergy extraction from the synergy-tuned task to extract a single synergy for each direction. Constraining the NMF output could potentially dismiss synergies necessary to perform the task (Al Borno et al. 2020). However, we found the same synergies, in number and structure, across those extracted from the multidirectional task and those from the synergy-tuned tasks. Third, clustering was also constrained to the maximum number of synergies across participants, and restricted to avoid repetition of synergies from the same participant as members of a single cluster. This decision conserves the separation made by the NMF analysis, but the original cluster membership is not conserved, making clusters more heterogeneous by reassigning memberships. Nevertheless, we decided to assign higher weight to the outputs of the NMF rather than clustering because NMF extracts the set of synergies from the original EMG signals. In contrast, clustering considers just a single component (structure) of synergies. Indeed, more research is needed regarding grouping techniques and their influence on the global analysis of synergies. Computational methods have now been developed that allow us to assess whether the extracted synergy clusters can perform the required tasks (Al Borno et al. 2020).

Similarly, our IMC results may be affected by the presence of cross-talk. We found IMC values above the threshold for all frequencies, suggesting cross-talk between the EMG channels. This possibility is more likely when the analysed muscles are closer to each other in the body, which is the case for some higher weight muscles within a synergy. However, despite high IMC across all synergies, the higher level IMC bands coincide with CSC significant frequencies. Moreover, if we remove the effects of cross-talk, a higher level of coherence in the mentioned band would still observed.

We have used in these analyses, a relatively recent method for calculating the significance threshold for coherence values, and there is no current standard to determine a significant level of coherence. It is possible that alternative methods would better estimate the coherence level under the null-hypothesis condition. Nonetheless, across our results, the same frequency bands exhibit a higher level of IMC and CSC, consistent with previous, independent findings. The observed correlation between EEG-EMG coherence and muscle synergies does not necessarily imply causality. While the coincidental emergence of correlated activity between signals is possible, and it is not possible to determine directionality, it is often the case of correlated activity being a sign of functional communication between structures. There is still uncertainty about the correlation between coherence frequency bands and the anatomical correlate of the signals. IMC and CMC seem sensitive to different descending pathways, where IMC is likely to be influenced by the reticulospinal tract (Boonstra et al. 2008) and CMC by the corticospinal tract (Boonstra 2009). CSC could be considered a recent finding; therefore, there is much to research about its anatomical correlates. Indeed, more research is needed, including non-linear methods (Yang et al. 2018), to clarify the relationship between IMC, CSC, and CMC changes and the neural substrate. Finally, other considerations are our small sample size, which limits the generalizability of the findings. Our focus was solely on isometric contractions of the upper limbs, where dynamic movements or other limbs may rely on different mechanisms. The usage of surface EMG to determine muscle activity does not capture deep muscle activity.

## Conclusion

The results, in agreement with other studies (Mayer et al. 2019; Muret and Makin 2021), suggest that long-range synchronous activity between the cortex, muscle synergies, and effectors is involved in generating complex behaviour through the coordinated and combinatorial recruitment of these representations in the motor cortex. The differential expression of IMC based on synergy structure, where higher weight muscles exhibit higher levels of coherence, implies a shared neural input responsible for producing muscle synergy. This shared neural input, as indicated by IMC’s α and β peaks, suggests that it arises from cortical and subcortical levels. However, this study does not rule out the possible existence of mechanically driven synergies. The functional connectivity that was observed within all synergies may suggest the potential for such mechanisms. Therefore, the current analysis paradigm and tools could be valuable in future investigations aimed at exploring this possibility. Along with IMC, the presence of CSC in the β band suggests closed-loop communication networks that modulate muscle synergies. The differential level of IMC between contributors within a synergy may reflect the level of co-regulation required to achieve the motor task. Given the observed shared neural input arising from cortical and subcortical levels, it can be suggested that synergies are a neural component of the CNS’s functional control strategy.

## Data Availability

Data can be made available from the corresponding author upon reasonable request.
